# Estrogen promotes fetal skeletal muscle mitochondrial distribution and ATP synthase activity important for insulin sensitivity in offspring

**DOI:** 10.1007/s12020-024-03764-w

**Published:** 2024-03-13

**Authors:** Soon Ok Kim, Eugene D. Albrecht, Gerald J. Pepe

**Affiliations:** 1 Department of Physiological Sciences, Eastern Virginia Medical School, Norfolk, VA USA; 2 Departments of Obstetrics/Gynecology/Reproductive Sciences and Physiology, University of Maryland School of Medicine, Baltimore, MD USA

**Keywords:** Estrogen, Insulin resistance, Skeletal muscle, Mitochondria, Fetus

## Abstract

**Purpose:**

We previously showed that offspring delivered to baboons in which levels of estradiol (E_2_) were suppressed during the second half of gestation exhibit insulin resistance. Mitochondria are essential for the production of ATP as the main source of energy for intracellular metabolic pathways, and skeletal muscle of type 2 diabetics exhibit mitochondrial abnormalities. Mitochondria express estrogen receptor β and E_2_ enhances mitochondrial function in adults. Therefore, the current study ascertained whether exposure of the fetus to E_2_ is essential for mitochondrial development.

**Methods:**

Levels of ATP synthase and citrate synthase and the morphology of mitochondria were determined in fetal skeletal muscle obtained near term from baboons untreated or treated daily with the aromatase inhibitor letrozole or letrozole plus E_2_.

**Results:**

Specific activity and amount of ATP synthase were 2-fold lower (*P* < 0.05) in mitochondria from skeletal muscle of E_2_ suppressed letrozole-treated fetuses and restored to normal by treatment with letrozole plus E_2_. Immunocytochemistry showed that in contrast to the punctate formation of mitochondria in myocytes of untreated and letrozole plus E_2_ treated animals, mitochondria appeared to be diffuse in myocytes of estrogen-suppressed fetuses. However, citrate synthase activity and levels of proteins that control mitochondrial fission/fusion were similar in estrogen replete and suppressed animals.

**Conclusion:**

We suggest that estrogen is essential for fetal skeletal muscle mitochondrial development and thus glucose homeostasis in adulthood.

## Introduction

Our laboratories have shown that baboon offspring born to mothers treated with the aromatase inhibitor letrozole and thus deprived of estrogen during the second half of gestation exhibited insulin resistance and a deficit in first phase insulin release [1–3]. Insulin resistance was not due to an impairment of fetal or offspring growth, nor to an alteration in adipose sensitivity to insulin [3, 4]. Based on these findings and the fact that skeletal muscle accounts for more than 70% of total insulin-directed glucose utilization [5, 6], we proposed that estrogen plays an important role in programming mechanisms in fetal skeletal muscle that underpin insulin action and glucose homeostasis in offspring [1, 3].

More recently, we showed that the number of capillaries and the capillary/skeletal muscle fiber ratio, which is important for delivery of substrates and insulin to myofibers [3], and the size of individual muscle fibers [7], were markedly lower in near term fetuses deprived of estrogen. Moreover, the impairment in the microvessel/muscle fiber ratio was sustained in offspring that subsequently developed systemic vascular dysfunction, including reduced vascular endothelial-mediated vasodilation and hypertension, in addition to insulin resistance [3]. Therefore, we proposed that the elevation in estrogen during the second half of primate pregnancy promotes systemic micro-vascularization, as well as growth of fetal muscle fibers, essential for insulin sensitivity and vascular homeostasis in adulthood.

It is well established that mitochondria are essential for energy production and cellular homeostasis. Mitochondria possess their own genome (mtDNA), which includes genes that comprise the respiratory chain enzyme complexes and coupling of oxidative phosphorylation/ ATP production and electron transfer which is controlled in part by the ATP synthase enzyme complex. Physiologic bioenergetics also is dependent on the maintenance of a normal number and distribution of mitochondria, which is coordinated by fission and fusion [8], processes regulated by a family of proteins, including the fission proteins fission-1 (Fis1) and dynamin-related protein 1 (Drp1), and the fusion proteins optic atrophy gene 1 (Opa1) and mitofusion (Mfn-2). Importantly, patients with insulin resistance/type 2 diabetes exhibit mitochondrial dysfunction in several tissues including skeletal muscle [9] in which there is an alteration in mitochondrial morphology [10, 11], impaired oxidative phosphorylation and downregulation of genes involved in mitochondrial biogenesis [12–14]. Moreover, mitochondrial dysfunction in insulin resistance/type 2 diabetes is associated with generation of oxidative stress [9], which is often not overcome by antioxidant enzymatic defense mechanisms [15]. Interestingly, mitochondrial defects are also a hallmark feature in cardiac muscle of experimental models of hypertension and are a potential therapeutic target for patients with hypertension [16].

Studies have also shown that in adult animals, estrogen via interaction with estrogen receptor (ER) α and/or ER β enhances various aspects of skeletal muscle mitochondrial function [17–19]. For example, muscle specific ERα null mice exhibit reduced rates of oxygen consumption and mtDNA replication, increased production of reactive oxygen species (ROS), and an alteration in mitochondrial morphology [17, 20, 21]. In addition, in several cells/tissues, estrogen enhanced the levels of mtDNA encoded genes, including subunit E of ATP synthase, via the transcription factor NRF-1 [18, 22, 23]. Mitochondria, including those in skeletal muscle, express ER β and activation of mitochondrial ER β increased mtDNA encoded transcription of respiratory chain enzyme complexes e.g., cytochrome-oxidase, ATP synthase, as well as mitochondrial biogenesis [18, 19, 24, 25]. However, although human fetal skeletal muscle expresses both ERα and β [26] whether skeletal muscle mitochondrial development in utero is influenced by exposure of the fetus to estrogen is unknown. Therefore, the current study determined whether the amount and/or activity of mitochondrial ATP synthase and the distribution of mitochondria are altered in fetal skeletal muscle of baboons deprived of estrogen in utero.

## Research design and methods

### Animals

Female baboons, originally obtained from the Southwest National Primate Research Center (San Antonio, TX) were housed individually in large primate cages in air-conditioned rooms and received standard primate chow (Harlan Primate Diet, Madison, WI), fresh fruit twice daily and water ad libitum. Females were paired with male baboons for 5 days at mid-cycle as estimated by menstrual cycle history, pregnancy confirmed by ultrasound and day 1 designated as the day preceding perineal deturgesence. Animals were cared for and used strictly in accordance with USDA regulations and the NIH Guide for the Care and Use of Laboratory Animals (8th edition). The present protocols were approved by the Institutional Animal Care and Use Committees of the University of Maryland School of Medicine and the Eastern Virginia Medical School.

Serum and samples of fetal skeletal muscle were obtained from a contemporaneous group of baboons as part of our ongoing studies [3, 7]. Briefly, pregnant baboons were either untreated (*n* = 11) or treated daily on day 100 to days 165–175 of gestation (term = 184 days) with the aromatase inhibitor letrozole (4,4′-[1,2,3-triazyol-1-yl-mehylene]-bis-benzonitrite; Novartis Pharma AG, Basel, Switzerland; 115 µg/kg bw/day, maternal sc injection in 1.0 ml sesame oil; *n* = 10) or with letrozole (115 µg/kg bw/day) plus estradiol benzoate (25 μg/kg bw/day on day 100 increasing to 115 μg/kg bw/day between days 120 and 165–175; 1.2 ml sesame oil; *n* = 5). On days 165–175, baboons were anesthetized with isoflurane and blood samples obtained from the maternal saphenous vein (5 ml) and the umbilical artery (2 ml) and fetuses delivered by cesarean section and euthanized by an IV injection of pentobarbital (100 mg/kg bw). Samples (5–10 mm^3^) of fetal vastus lateralis skeletal muscle were placed in formalin and paraffin-embedded or snap frozen and stored in liquid nitrogen.

### Analyses

#### Determination of estradiol, insulin and glucose

Serum levels of estradiol in maternal saphenous vein and serum estradiol, plasma insulin and blood glucose in umbilical artery at delivery were determined using an automated chemiluminescent immunoassay system (Immulite; Siemens Healthcare Diagnostics, Deerfield, IL) and an iStat Portable Clinical Analyzer (glucose; Model #210003, Abbott Labs, East Windsor, NJ) as described previously [1, 27].

#### ATP synthase activity and content

The activity and content of ATP synthase (EC 3.6.3.14; Complex V) were determined using a multiplexing microplate kit, and procedures and reagents provided by the manufacturer (Abcam, Cambridge, MA). Fetal skeletal muscle (~100 mg) from baboons untreated (*n* = 6; 4 ♂; 2 ♀) or treated with letrozole (*n* = 6; 5 ♂; 1 ♀), or letrozole plus estradiol benzoate (*n* = 3; 2 ♂; 1 ♀) were homogenized in phosphate-buffered saline (PBS), centrifuged (16,000 rpm) and the pellet containing an enriched fraction of mitochondria resuspended in buffer and the protein concentration (bicinchoninic acid; Sigma Aldrich, St Louis, MO) adjusted to 5.5 mg/ml. Detergent supplied by the manufacturer was added to solubilize and obtain intact ATP synthase protein, which was immuno-captured onto microplate wells (40 µg protein/well). Samples were incubated (30 C) and ATP synthase activity, which was coupled to the oxidation of NADH to NAD^+^ by lactate dehydrogenase in the reaction buffer, determined by measuring the decrease in NADH absorbance (340 nm) for 120 min using a Spectra Max multimode microplate reader (Molecular Devices, Sunnyvale, CA). After completion of these analyses, an ATP synthase specific antibody conjugated with alkaline phosphatase and a proprietary substrate provided by the manufacturer was added to the wells. The colorless substrate is converted to a yellow product by alkaline phosphatase and thus the quantity of ATP synthase is proportional to the activity of alkaline phosphatase. Accordingly, ATP synthase content was determined by measuring the increase in absorbance at 405 nm over a 30 min incubation (room temperature) period using the Spectra Max multimode microplate reader. A null buffer to estimate non-specific background was analyzed concomitantly. ATP synthase activity and quantity were expressed as change in absorbance at 340 and 405 nm/min/µg protein, respectively.

#### Citrate synthase activity

Citrate synthase activity was determined in mitochondria from baboons untreated (*n* = 7; 5 ♂, 2 ♀) or treated with letrozole (*n* = 7; 5 ♂, 2 ♀) or letrozole + estradiol benzoate (*n* = 3; 2 ♂, 1 ♀) using an assay kit, reagents and instructions provided by the manufacturer (Cayman Chemical, Ann Arbor, MI). Aliquots of mitochondrial protein (5.5 mg protein/ml) as well as protein from a control tissue provided by the manufacturer were added to a 96 well microplate to which was added buffer containing acetyl-CoA and 5,5′-di-thiobis-(2-nitrobenzoic acid). After addition of oxaloacetate (time 0), citrate synthase activity was determined (room temperature) by measuring the change in absorbance at 412 nm over a 20 min period using the Spectra Max multimode microplate reader.

#### Morphology of mitochondria

Paraffin sections (5 μm; 3–4 per animal) of fetal vastus lateralis from baboons untreated (*n* = 4) or treated with letrozole (*n* = 4) or letrozole + estradiol benzoate (*n* = 3) were boiled in 0.01 M sodium citrate buffer (pH 6.0), incubated with Image-iT signal enhancer (Molecular Probes Inc., Eugene, OR), blocked with 5% normal goat serum (NGS; Vector Laboratories, Burlingame, CA) in PBS and treated with streptavidin/biotin blocking reagent according to the manufacturer (Vector). Sections were then incubated (4 C) overnight with primary anti-mitochondria antibody (Table 1) diluted 1:250 in 5% NGS. After washing with PBS, sections were successively incubated with biotinylated goat anti-mouse secondary antibody (BA-9200, RRID AB 2336171; 1:200 dilution; Vector), streptavidin conjugated with AlexaFluor 488 Green (Molecular Probes) diluted 1:500 in 5% NGS, Sudan Black (1% in 70% methanol) to quench autofluorescence, and propidium iodide (0.2 μg/ml PBS) to stain nuclei red. After application of Prolong Gold Antifade Reagent (Molecular Probes), slides were sealed with nail polish and stored in the dark (4C) until examined using an Olympus BX41 microscope (Optical Elements Corp., Melville, NY) equipped with an Olympus DP70 digital camera with FITC/TRITC filter sets.

**Table 1:** Antibodies

Protein Target	Manufacturer Catalog Number	RRID	Protein	Dilution
Catalase	AB 179843	AB 2716714	Rabbit Monoclonal	1:250
Superoxide dismutase	AB 179843	AB 2716714	Rabbit Monoclonal	1:250
Thioredoxin	AB 179843	AB 2716714	Rabbit Monoclonal	1:250
Peroxiredoxin 3	AB 73349	AB 1860862	Rabbit Polyclonal	1:1000
Dynamin-related Protein 1	AB 180769	AB 2924856	Rabbit Polyclonal	1:500
Mitofusion 2	AB 101055	AB 2924857	Rabbit Polyclonal	1:1000
Fission Protein 1	AB 156865	AB 2924858	Rabbit Monoclonal	1:2000
Optic Atrophy Gene 1	CS 67589	AB 2799728	Rabbit Monoclonal	1:1000
Mitochondria	AB 3298	AB 303683	Mouse Monoclonal	1:250
GAPDH	AB 9385	AB 449791	Rabbit Polyclonal	1:5000

*AB* Abcam, *CS* Cell Signaling

#### Western blot analyses

The expression of proteins controlling mitochondrial fission (Fis1, Drp1) and fusion (Opa1, Mfn-2) and the antioxidant enzymes catalase, superoxide dismutase (SOD1), thioredoxin (THX) and mitochondrial peroxiredoxin 3 (PRX3) was determined by Western blot using primary antibodies (Table 1) and procedures described previously [1, 2]. Briefly, samples were homogenized on ice in PBS with 1% cholic acid, 0.1% SDS, 1 mM EDTA, and a protease/ phosphatase inhibitor cocktail. After determination of protein concentrations (bicinchoninic acid method; Sigma), proteins (20 µg) were separated by SDS-polyacrylamide gels and transferred onto an Immobilon-P membrane (Millipore Corp., Bedford, MA). Membranes were blocked 1 h at room temperature with 5% BSA in TBST (10 mM Tris-HCl, pH 7.5, 150 mM NaCl and 0.2% Tween 20), and then incubated (overnight; 4 C) with respective primary antibody. After washing with TBST, membranes were incubated (1 h; room temperature) with horseradish peroxidase (HRP)-labeled secondary antibody (horse anti-mouse IgG; catalog PI-2000; RRID AB 2336177; goat anti-rabbit IgG; catalog PI-1000; RRID AB 2336198; Vector) diluted 1:5000 in TBST + 5% BSA. Membranes were washed with TBST, developed with enhanced chemiluminescence (GE Healthcare, Pittsburgh, PA) and band intensities on Fuji Super RX medical x-ray film (Fujifilm Medical Systems, USA, Inc., Roselle, IL) quantified by densitometry using Image J software (NIH). Blots were then stripped and re-probed using HRP-conjugated GAPDH (Table 1) diluted 1:5000 and results (arbitrary densitometric units/µg protein) expressed as a ratio to GAPDH. Specificity of primary antibodies was determined by incubation of samples without primary antibody.

### Statistical analyses

Although the number of skeletal muscle samples from male fetuses exceeded that from females, the apparent distribution and individual values for various parameters were similar in males and females. Accordingly, values were combined and expressed as an overall mean ± SEM and analyzed by One-Way Analysis of Variance (ANOVA) and post hoc comparison of the means using the Student-Newman Keuls statistic and GraphPad InStat (La Jolla, CA).

### Data and resource availability

All data generated and analyzed are included in the published article and the RRIDs for antibodies employed outlined in the text and/or Table 1.

## Results

### Maternal estradiol and fetal estradiol, insulin and glucose levels and body and placental weights

The levels of estradiol in maternal serum of baboons untreated or treated with letrozole ± estradiol benzoate between days 100 and 165–175 of gestation and in maternal and umbilical artery at the time of delivery (Table 2) have been previously published [1, 7]. Thus, as previously described [7], in contrast to the progressive increase in maternal estradiol levels during the second half of gestation in untreated baboons, administration of letrozole rapidly decreased maternal estradiol to levels <7% of that in untreated animals (*P* < 0.001). The pattern and absolute levels of estradiol were restored to normal by maternal administration of letrozole and estradiol benzoate. Similarly, the level of estradiol in the umbilical artery (0.03 ± 0.01 ng/ml) in letrozole-treated animals was lower (*P* < 0.001) than in untreated animals (0.77 ± 0.12 ng/ml). However, although letrozole plus estradiol benzoate treatment increased (*P* < 0.01) umbilical artery estradiol to a value (0.08 ± 0.01 ng/ml) greater than that in animals treated with letrozole alone, levels were lower (*P* < 0.001) than in untreated baboons (Table 2), presumably due to placental metabolism of maternally administered estradiol.

**Table 2:** Levels of estradiol (E_2_) in maternal saphenous (MS) vein, of E_2_, glucose, and insulin in umbilical artery and placental and fetal body weights in late gestation in baboons^a^

Treatment	MS	Umbilical Artery			Weight	
	E_2_ ng/ml	E_2_ ng/ml	Glucose (mg/dl)	Insulin (IU/ml)	Placenta (gm)	Body (gm)
Untreated	3.07 ± 0.13^b^	0.77 ± 0.12^b^	74 ± 8	5.0 ± 1.3	201 ± 11	898 ± 25
Letrozole	0.29 ± 0.06^c^	0.03 ± 0.01^c^	76 ± 3	4.2 ± 1.4	220 ± 14	932 ± 19
Letrozole+E_2_	3.47 ± 0.11^b^	0.08 ± 0.01^d^	72 ± 3	7.0 ± 2.2	206 ± 10	914 ± 34

^a^Values expressed as Mean ± SEM on the day of delivery (day 165–175) in baboons untreated (*n* = 11) or treated via maternal sc injection on days 100 to 165–175 (term = 184 days) with letrozole (115 µg/kg bw/day; *n* = 10) or letrozole (115 µg/kg bw/day) plus estradiol benzoate (25 μg/kg bw/day on day 100 increasing to 115 μg/kg bw/day between days 120 to 165–175; *n* = 5)

^b,c,d^Values with different letter superscripts differ at *P* < 0.01–<0.001 (maternal saphenous) and *P* < 0.05 - <0.01 (umbilical artery). One-Way ANOVA; *P* < 0.0001 maternal saphenous and *P* < 0.001 umbilical artery; and Tukey-HSD/Kramer post tests. E_2_ values in umbilical artery log transformed

Data from Kim et al. (Ref. [7])

In contrast, as shown previously [1, 7] umbilical artery plasma insulin and blood glucose levels as well as placental and fetal body weights, were similar in baboons untreated or treated with letrozole ± estradiol benzoate (Table 2).

### ATP synthase activity and content

**Fig. 1:**
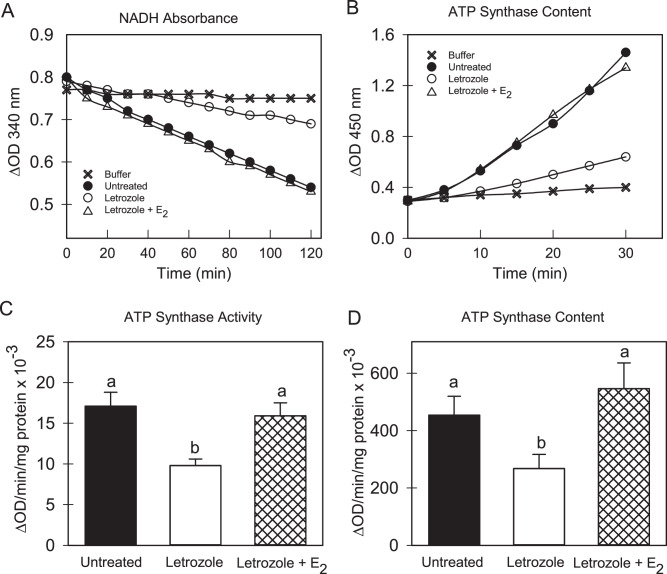
Representative examples of the decrease in absorbance of NADH reflective of ATP synthase activity (**A**) and the increase in conversion of a proprietary substrate to and absorbance of a yellow product catalyzed by alkaline phosphatase-linked to the content/amount of ATP synthase protein (**B**) in fetal skeletal muscle mitochondria on day 165–175 of gestation (term = day 184) in baboons untreated or treated with letrozole or letrozole plus estradiol benzoate (E_2_) compared to buffer alone. The overall mean (±SEM) specific activity of ATP synthase (**C**) and amount of ATP synthase protein (**D**) in enriched fractions of mitochondria in fetal skeletal muscle obtained on days 165–175 of gestation from baboons untreated (*n* = 6) or treated with letrozole (*n* = 6) or letrozole plus E_2_ (*n* = 3) as described in legend to Table 2. Enzyme analyses corrected for change in absorbance in wells incubated with buffer alone. Values with different letter superscripts differ at *P* < 0.05–0.01 (One Way ANOVA, *P* = 0.0046 for difference in ATP synthase activity; *P* = 0.0351 for difference in ATP synthase content and Student-Newman Keuls Multiple Comparison Statistic)

Figure 1A depicts representative examples of the time-dependent decrease in NADH levels/decrease in absorbance reflecting the specific activity of ATP synthase in fetal skeletal muscle mitochondria from baboons untreated or treated with letrozole ± estradiol benzoate. As seen, oxidation of NADH to NAD^+^ and thus ATP synthase activity was linear over the range of time points examined and specificity confirmed by minimal change in absorbance in reactions without fetal tissue extracts. Moreover, enzyme activity appeared to be greater in mitochondria from untreated vs letrozole-treated baboons and restored by treatment with estrogen. Thus, as shown in Fig. 1C, the overall mean (±SEM) level of ATP synthase activity (ΔOD/min/mg protein × 10^−3^) in mitochondria of untreated animals (17.1 ± 1.7) was reduced approximately 40% (*P* < 0.05) in estrogen-suppressed animals (9.8 ± 0.8) and restored (*P* < 0.05) to normal by treatment with letrozole and estradiol benzoate (16.0 ± 0.9).

Figure 1B depicts representative examples of the increase in conversion of a proprietary substrate to a yellow product by alkaline phosphatase linked to ATP synthase protein present in the reaction mixture following immunoprecipitation of the enzyme from fetal skeletal muscle of untreated and letrozole ± estradiol benzoate-treated baboons. As seen, the change in absorbance, which is reflective of the amount of ATP synthase, was linear over the time period examined and specificity confirmed by minimal change in absorbance in reaction mixtures to which fetal tissue extracts were not added. Moreover, the amount of ATP synthase appeared to be greater in mitochondria of untreated vs letrozole-treated baboons. Thus, as shown in Fig. 1D, the overall mean (±SEM) content of ATP synthase (ΔOD/min/mg protein × 10^−3^) in mitochondria of untreated animals (455 ± 65) was approximately 40% lower (*P* < 0.05) in mitochondria of animals treated with letrozole (268 ± 49) and restored to normal (*P* < 0.05) by treatment with letrozole + estradiol benzoate (546 ± 89).

However, the ratio of ATP synthase activity: ATP synthase content (x100) was similar in mitochondria in fetal skeletal muscle of baboons untreated (4.0 ± 0.5), treated with letrozole (4.3 ± 0.8) or with letrozole + estradiol benzoate (4.6 ± 0.3).

**Fig. 2:**
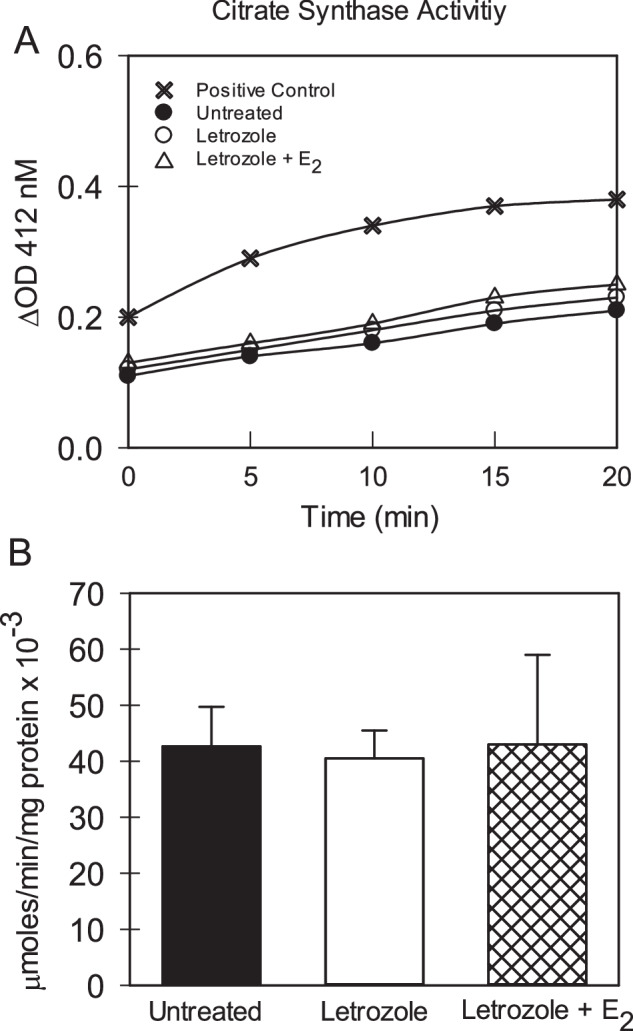
**A** Representative examples of the increase in absorbance and thus formation of thio-nitro-benzoic acid catalyzed by citrate synthase in enriched fractions of mitochondria in fetal skeletal muscle on day 165–175 of gestation from baboons untreated or treated with letrozole ± estradiol benzoate (E_2_) and in a sample of manufacturer-supplied tissue control. **B** Overall mean (±SEM) level of citrate synthase specific activity in fetal skeletal muscle mitochondria on day 165–175 of gestation (term = day 184) in baboons untreated (*n* = 7) or treated with letrozole (*n* = 7) or letrozole plus E_2_ (*n* = 3). Enzyme analyses corrected for change in absorbance in wells incubated with buffer alone (not shown)

Figure 2A depicts representative examples of citrate synthase dependent formation of thio-nitro-benzoic acid by mitochondria from fetal skeletal muscle of baboons untreated or treated with letrozole ± estradiol benzoate. As seen, the rate of formation of product the amount (ΔOD) of which is proportional to the activity of citrate synthase was linear over the time period examined. Moreover, enzyme activity was detectable at the expected level in a control tissue supplied by the manufacturer. As seen in Fig. 2B, citrate synthase specific activity (µmoles/min/mg protein × 10^−3^) was similar in mitochondria from fetal skeletal muscle of baboons untreated (42 ± 8) or treated with letrozole (40 ± 4) or letrozole + estradiol benzoate (42 ± 16).

### Mitochondrial dynamics in fetal skeletal muscle

**Fig. 3:**
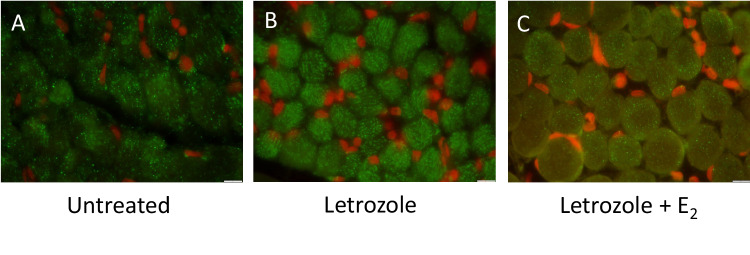
Representative sections of the immunocytochemical expression of the 60 kDa non glycosylated mitochondrial component/protein (green) to ascertain the cellular localization/ distribution of mitochondria in paraffin embedded sections of fetal skeletal muscle obtained on day 165–175 of gestation from baboons (**A**) untreated (*n* = 4), (**B**) treated with letrozole (*n* = 4), or (**C**) treated with letrozole plus E_2_ (*n* = 3) as outlined in legend to Table 2. Red, myocyte nuclei. Original magnification 1000X. Scale bar (10 µm) bottom right

In fetal skeletal muscle of untreated baboons, the immunocytochemical expression of the 60 kDa mitochondrial protein was punctate and cellular distribution appeared to be organized and fairly uniform throughout the myocytes (Fig. 3A). In contrast, in estrogen-suppressed animals, mitochondrial 60-kDa protein expression appeared to be more diffuse suggesting that the morphology of mitochondria was altered within fetal myocytes (Fig. 3B). In letrozole plus estradiol benzoate-treated animals, mitochondrial 60 kDa protein expression and thus morphology of mitochondria was restored to normal in the majority of myocytes (Fig. 3C).

**Fig. 4:**
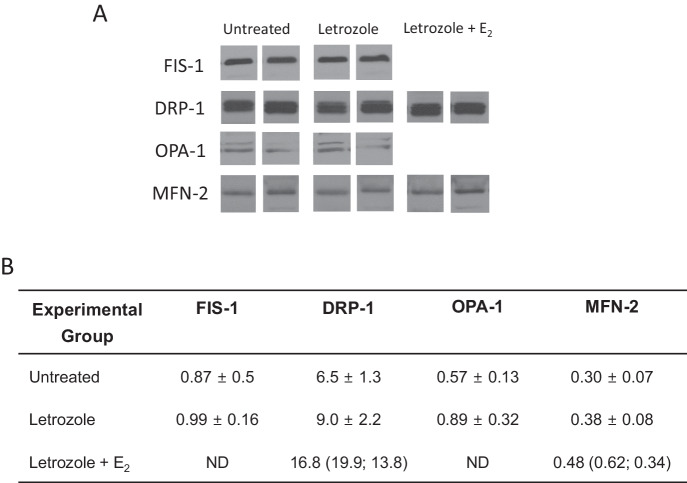
**A** Representative Western immunoblot of fission protein 1 (Fis-1), dynamin-related protein-1 (Drp-1), optic atrophy gene-1 (Opa-1) and mitofusion 2 (Mfn-2) protein expression in fetal skeletal muscle on day 165–175 of gestation in baboons untreated or treated with letrozole or letrozole plus estradiol benzoate (E_2_). Fis-1, Drp-1, Opa-1 and Mfn-2 electrophoresed on separate membranes and protein bands from representative samples cropped and positioned for illustrative purposes. Specificity of the primary antibodies was confirmed by absence of signal in samples incubated without primary antibody. **B** Mean ± SEM levels of fission (Fis1, Drp1) and fusion (Opa-1; Mfn-2) proteins (expressed as ratio to GAPDH) in fetal skeletal muscle on day 165–175 of gestation in baboons untreated (*n* = 8; *n* = 4 Fis-1), or treated with letrozole (*n* = 8; *n* = 4 Fis-1) or letrozole plus E_2_ (*n* = 2). ND not determined

**Fig. 5:**
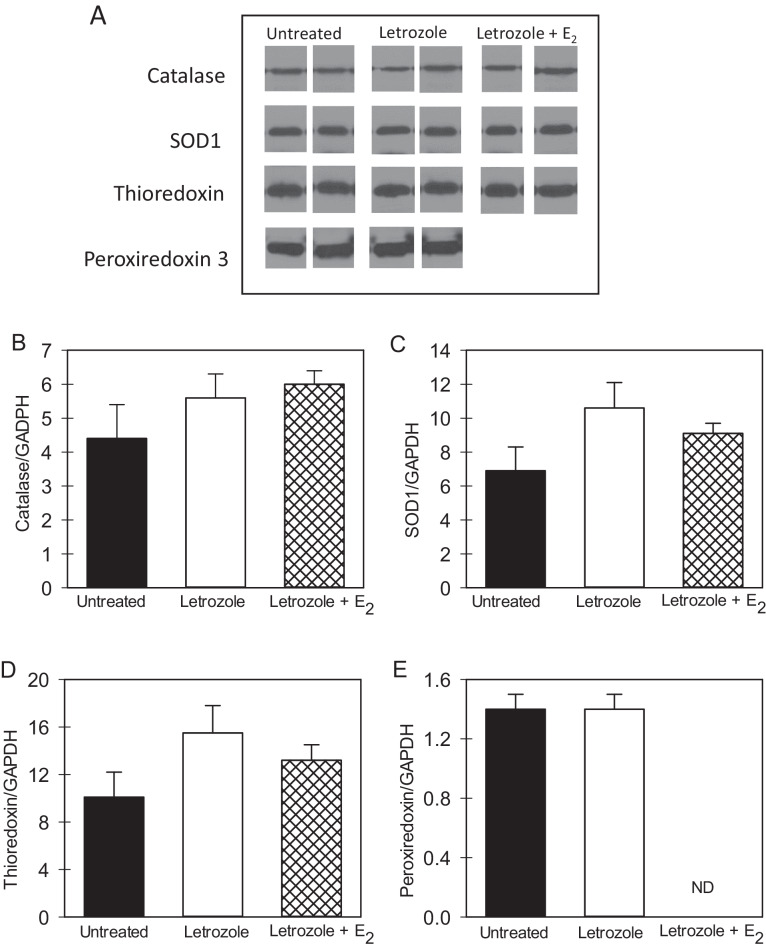
**A** Representative Western immunoblot of catalase, superoxide dismutase (SOD-1), thioredoxin, peroxiredoxin 3 protein expression in fetal skeletal muscle on day 165–175 of gestation in baboons untreated or treated with letrozole or letrozole plus estradiol benzoate (E_2_). Catalase, SOD1 and TRX electrophoresed on the same membrane and periredoxin 3 on a separate membrane and protein bands from representative samples cropped and positioned for illustrative purposes. Specificity of the primary antibodies was confirmed by absence of signal in samples incubated without primary antibody. **B**–**E** Mean (±SEM) protein levels (ratio to GAPDH) of catalase, SOD 1, thioredoxin and peroxiredoxin 3 in fetal skeletal muscle obtained on days 165–175 of gestation from baboons untreated (*n* = 8; *n* = 4 peroxiredoxin 3) or treated with letrozole (*n* = 8; *n* = 4 peroxiredoxin 3) or letrozole plus E_2_ (*n* = 2; peroxiredoxin 3 not determined, ND)

The dynamin proteins that regulate mitochondrial fission (Fis1 and Drp1) and fusion (Opa1 and Mfn-2) were detected by Western blot and exhibited a molecular size of 17, 82, 80–100 and 77 kDa, respectively (Fig. 4A). As shown in Fig. 4B, the overall mean levels (relative to GAPDH) of Fis1, Drp1, Opa1, and Mfn-2 were similar in fetal skeletal muscle of untreated and letrozole ± estradiol benzoate-treated baboons. Moreover, the mean levels (relative to GAPDH) of the antioxidant enzymes catalase, SOD1, THX and mitochondrial PRX3, which exhibited molecular sizes of 60, 16, 12, and 23 kDa, respectively (Fig. 5A), were similar in fetal skeletal muscle of untreated and letrozole ± estradiol benzoate-treated baboons (Fig. 5B).

## Discussion

The results of the current study show that the specific activity and content of ATP synthase, which is critical for the production of ATP, the major source of energy for intracellular metabolic pathways, were markedly reduced in mitochondria from skeletal muscle of estrogen suppressed fetuses compared to respective levels in mitochondria of untreated animals exposed to the normal increase in estrogen with advancing gestation. The decrease in ATP synthase did not reflect an alteration in the levels of glucose or insulin in fetal blood and thus potentially available to muscle cells. Importantly, the ratio of ATP synthase activity to enzyme content was similar in mitochondria of untreated and letrozole-treated animals. Thus, the decrease in ATP synthase activity in skeletal muscle mitochondria of estrogen-suppressed fetuses appears to reflect a decrease in the amount of enzyme synthesized and not the result of a chemical modification, catalytic point mutation or uncoupling of the F1 and F0 domains of the enzyme complex. Whether other enzymes in the electron transport chain were modified remains to be determined. However, the current study showed that fetal skeletal muscle mitochondrial citrate synthase activity was not altered by estrogen supporting specificity in the action of estradiol. Importantly, the levels of ATP synthase activity and content were restored to normal in fetal skeletal muscle of baboons treated with letrozole plus estradiol benzoate. Collectively, these results indicate that exposure of the fetus to estrogen during the second half of gestation is important for the synthesis and the specific activity of the ATP synthase enzyme complex reflecting mitochondrial function within fetal skeletal muscle.

Despite the marked reduction in the content/specific activity of ATP synthase in mitochondria of estrogen-deprived fetuses, it remains to be determined whether mitochondrial ATP levels as well as basal and/or maximal oxygen consumption rates are also compromised. Homeostatic mitochondrial bioenergetics, e.g., ATP synthesis, is dependent upon the maintenance of normal mitochondrial number, distribution and morphology [8, 28]. As shown in the present study, although quantitative analyses were not performed, the morphology and organization of mitochondria appeared to be altered in fetal skeletal muscle of estrogen-deprived animals. Therefore, it is possible but remains to be determined that the decline in ATP synthase in fetal skeletal muscle mitochondria of estrogen-suppressed baboons, along with the alteration in myocyte mitochondrial morphology, result in a reduction in ATP synthesis and other aspects of mitochondrial function. The latter changes in fetal mitochondria, could underpin the onset of insulin insensitivity exhibited in offspring delivered to estrogen-deprived baboons. Consistent with this suggestion, abnormalities in mitochondrial function, including deficiency in the electron transport chain enzymes, are apparent in skeletal muscle of patients with insulin resistance/type 2 diabetes, a deficiency which also correlates with the severity of insulin-resistant glucose metabolism [10, 12, 29, 30]. Moreover, insufficient mitochondrial ATP biogenesis compromises the activity of additional key enzymes, e.g., hexokinase, requiring phosphorylation to support glucose metabolism [10, 31].

It is well established that the distribution and number of mitochondria is controlled by fission and fusion, two opposing processes highly coordinated by GTPases in the dynamin family [8], including the fission proteins Fis1 and Drp1 and the fusion proteins Opa1 and Mfn-2 [8, 32]. Expression of Mfn2 was reduced in skeletal muscle of obese and lean type 2 diabetic patients compared to lean insulin sensitive individuals [33, 34]. Moreover, expression of Fis1 and Drp1 was elevated in skeletal muscle of genetic- and diet-induced obese insulin insensitive mice, and associated with a decrease in ATP production and insulin-stimulated glucose uptake, which was restored by treatment with a Drp1 inhibitor [30, 35]. Although the factors controlling expression of these proteins in baboon fetal skeletal muscle remain to be determined, estrogen has been shown in adult animals to regulate mitochondrial dynamics, promote fusion and attenuate fission by controlling expression of requisite fission/fusion proteins ([25] for review). However, despite the change in organization of myocyte mitochondria in estrogen-deprived fetuses of the current study, expression of proteins controlling fission (Fis1 and Drp1) and fusion (Opa1 and MIf-2) were not altered by estrogen and must be regulated by other factors.

Recently we showed that the number of microvessels and the microvessel/myofiber ratio, important for delivery of insulin and glucose to cells, was markedly reduced in fetal skeletal muscle of baboons deprived of estrogen by treatment with letrozole and restored to normal by concomitant treatment with letrozole plus estradiol [3]. Therefore, the alteration in mitochondrial morphology and decrease in ATP synthase may result from impairment in microvascular development induced by estrogen deprivation. Alternatively, and/or in addition, because ER α and β are expressed in fetal as well as adult skeletal muscle [26, 36, 37] and ERβ also localized to mitochondria [38, 39], it is possible that alterations in fetal skeletal muscle mitochondria observed in the current study reflect a direct action of estrogen. Although additional studies are required to ascertain the latter, muscle specific ERα null mice exhibit reduced rates of oxygen consumption and mtDNA replication, decreased energy expenditure and abnormal glucose homeostasis [25, 40] as well as altered mitochondrial morphology i.e., fission-fusion [17, 20, 21]. In addition, estrogen via activation of ERα and ERβ and the transcription factor NRF-1 upregulated the levels of mtDNA encoded genes including subunit E of ATP synthase [18, 22, 23]. Administration of estrogen to adult animals also enhanced skeletal muscle ATP production, the generation of mitochondrial membrane potential, and mitochondrial biogenesis [20, 25, 41, 42].

It also remains to be determined whether the reduction in ATP synthase and alteration in the morphology of mitochondria in skeletal muscle of estrogen-suppressed fetuses of the current study is accompanied by induction of oxidative stress. The latter seems unlikely, however, since the placenta plays a significant role in eliminating fetal waste products and providing maternal nutrients and other factors critical for fetal homeostasis [43]. Moreover, the levels of four important antioxidant enzymes including mitochondrial peroxiredoxin 3, were comparable in skeletal muscle of estrogen-replete and suppressed baboons. However, since offspring of estrogen-suppressed baboons develop insulin resistance early in postnatal life, it is possible, but remains to be determined whether the alteration in skeletal muscle mitochondria in fetal skeletal muscle of estrogen-deficient baboons is sustained in offspring and accompanied by local oxidative stress and/or an alteration in antioxidant enzyme systems.

In summary, the current study showed that the specific activity and amount of ATP synthase were lower in mitochondria from skeletal muscle of estradiol-suppressed fetuses and restored to normal by concomitant treatment with letrozole plus estradiol. Moreover, in contrast to the punctate and fairly uniform organization of mitochondria in myocytes of estrogen replete animals, mitochondria appeared to be more diffuse in myocytes of estrogen-suppressed fetuses. However, citrate synthase activity and levels of proteins that control mitochondrial fission and fusion were similar in fetal skeletal muscle of estrogen replete and suppressed animals. Based on these results and our previous studies, we suggest that estrogen is essential for skeletal muscle mitochondrial development and thus insulin sensitivity - glucose homeostasis in adulthood.
